# Effects of donor age and proliferative aging on the phenotype stability of rat aortic smooth muscle cells

**DOI:** 10.14814/phy2.12626

**Published:** 2015-11-24

**Authors:** Ana Martín-Pardillos, Víctor Sorribas

**Affiliations:** Department of Toxicology, University of ZaragozaZaragoza, Spain

**Keywords:** Aging, proliferation, senescence, vascular calcification, vascular smooth muscle cells

## Abstract

Age-related effects of the vascular wall have been associated with several hemodynamic dysfunctions, including medial vascular calcification. Vascular aging has been traditionally addressed using proliferative senescence of vascular smooth muscle cells (VSMC) in vitro, which induces osteoblastic transition and favors calcification in vitro. In this work, we have analyzed the relationship between organismal aging and proliferative senescence by comparing the proliferative aging of VSMC obtained from young, mature, and old rats (2-, 12-, and 24-month cell lines [CL], respectively). VSMC proliferated to more than 100 cumulative population doublings (CPD) without evidence of proliferative senescence, most likely as a consequence of telomerase induction. The apoptosis rate increased with CPD in all three CL, but the oxidation status of the cells was not modified. The magnitude of all gene expression changes caused by CPD was higher than the magnitude of the changes caused by donor age: the expressions of VSMC markers *α*-actin and SM22*α* decreased, while the expressions of transcription factors Msx2 and Runx2 and of bone morphogenetic protein-2 increased. Treatment of the cells with 2 mmol/L Pi revealed that the intensity of the effect of CPD on calcium deposition was greater than the effect of donor age. In conclusion, the proliferative lifespan of VSMC magnifies the effect of donor age on the osteoblastic transition of VSMC, therefore suggesting that in vivo vascular aging changes can be less dramatic than what is shown by in vitro aging.

## Introduction

Aging is a multifactorial, degenerative process that affects all animal species, and it is disclosed when individuals are protected from environmental hazards such as predation, accidents, starvation, or disease. The immediate consequence of this protection is increased survival, which according to the disposable soma theory (Kirkwood 1977) causes the progressive depletion (i.e., aging) of species specific, functional, metabolic, and repair reserves when the reproductive age is exceeded. At a cellular scale, aging affects both postmitotic and proliferative cells, with different consequences. In proliferative cells, senescence refers to the irreversible arrest of cell division in vitro when the Hayflick limit is reached after a progressive slowing down of cell division (Hayflick 1965). While senescence is optimistically interpreted as an oncosuppressive mechanism, antagonistic pleiotropy also reveals the negative consequences of senescence in organismal aging: the progressive loss of proliferative capacity is also accompanied, at the postreproductive age, by the genomic instability of cells, telomere shortening, epigenetic changes, and phenotype alterations such as secretory transformation (Campisi 2013; López-Otín et al. 2013).

Since the discovery of senescent cells in vivo (Dimri et al. 1995), the accumulation of these cells in tissues has been associated with many age-related degenerative processes (Neves et al. 2015). With respect to vascular biology, the implication of endothelial and smooth muscle cell senescence in atherosclerosis and vascular calcification (VC) has been intensely debated over the last decade (Minamino et al. 2002; Burton et al. 2009)^.^ Our present work focuses on the effect of senescence on VC. This ectopic calcium deposition can affect either the intima (prior to atheroma) or the media layer of arteries. Medial VC is frequently found in chronic kidney disease (CKD) that occurs with hyperphosphatemia and uremic “toxins,” but the prevalence is highest in diabetes, in addition to aging (Lanzer et al. 2014). Contrary to the physiological calcification of bones, the mineralization of arteries causes harmful effects derived from hardening of the wall, such as hypertension, stiffening, accelerated pulse, deficient peripheral perfusion, left ventricular overload and dysfunction, etc. (Lanzer et al. 2014). Deposits of calcium phosphates are mainly found on elastin fibers, and vascular smooth muscle cells (VSMC) are known to partially *trans*differentiate in bone-forming cells that express osteogenes, such as Runx2, Msx2, Bmp2, tissue-nonspecific alkaline phosphatase (TNAP), etc. (Chen et al. 2006). However, the pathogenesis of VC is not a common route in all instances. In the case of CKD, for example, uremic toxins seem to be a key causative factor in the transdifferentiation of VSMC, because among other effects, they increase TNAP expression (Chen et al. 2006). Calcium phosphates are most likely nucleated on elastic fibers and on VSMC and are endocytosed, causing cell death (Ewence et al. 2008), which creates additional nucleation sites and calcium deposition. Other suggested mechanisms that are involved in VC include autophagia (Dai et al. 2013), exosome secretion (Kapustin et al. 2015), oxidative stress (Byon et al. 2008), etc. During aging, however, VC mainly occurs in the absence of uremic toxins or hyperphosphatemia, but it contributes to the high morbidity and fragility of the elderly (Dao et al. 2005). VSMC senescence and telomere shortening have been associated with an increased severity of atherosclerosis (Matthews et al. 2006). When VSMC senescence is forced in vitro, the consequent phenotype predisposes the cells to calcification, with the expression of several genes that are necessary for osteoblast function, inflammation, and tissue remodeling, as determined by microarray screening and quantitative PCR (Nakano-Kurimoto et al. 2009; Burton et al. 2010). In addition, nuclear lamina dysfunction has also been described in senescent VSMC (Ragnauth et al. 2010). These cells accumulate the precursor prelamin A, thereby causing mitosis disruption and DNA damage, which also increases VC through the secretion of pro-osteogenic factors (Liu et al. 2013).

While most studies on VSMC have been performed in vitro, senescence markers such as *β*-galactosidase and p16 expression have also been observed in vivo in aged rats (Yang et al. 2007). This information therefore suggests that the senescence-associated *trans*differentiation of VSMC in vivo could be a major pathogenetic mechanism of VC in the elderly. Given that the proliferative potential (and therefore the in vitro senescence) of VSMC is also dependent upon the age of the human donor (Ruiz-Torres et al. 1999), in this work, we aimed to compare the chronological (in vivo) and proliferative (in vitro) aging of the cells and the likely effects on VC predisposition. Considering that the experimental culture conditions can affect the aging of cells very differently than what occurs during slow aging in vivo, our objective was to learn if the phenotypic changes that are observed during senescence in vitro and that predispose to ectopic VC are also observed during the aging of cells in vivo. To do this, we obtained primary cultures from 2-, 12-, and 24-month-old rats, and we studied the conservation of the smooth muscle contractile phenotype at different cumulative population doublings (CPD). Simultaneously, we analyzed the expression of a putative calcification phenotype along the culture life of the three cell lines (CL), and the findings were correlated with a molecular characterization of senescence in vitro and of proliferative lifespan. The final conclusion is that the phenotypic changes observed during proliferative aging in vitro are similar to the changes observed in vivo, but they are of a greater magnitude and are exaggerated by the experimental conditions in vitro.

## Methods

### Primary cultures and animals

Vascular smooth muscle cells were obtained by collagenase I digestion from the aortas of male Wistar rats (Janvier SAS, Saint-Berthevin, France) of 2, 12, and 24 months of age, with eight rats per age group, as published (Chamley et al. 1977). All studies on animals were conducted in accordance with the European legislation and were previously approved by the Ethical Committee of the University of Zaragoza. Rats were kept with free access to food and water, and after deep anesthesia with pentobarbital, the aortas were obtained and immediately processed. VSMC were grown in minimum essential medium (MEM) with 10% fetal calf serum, glutamine, and antibiotics. For the various assays, when the cells reached 90% confluence, they were made quiescent with 0.2% FCS-containing medium, unless otherwise specified. All culture reagents were from Gibco (Paisley, UK).

### Cell culture growth assay

The analysis of the growth and multiplication of the three VSMC cultures began after 17 divisions once the primary cultures had been initiated. For every passage, the cells were trypsinized, counted, and inoculated in a new flask at 1 × 10^4^ cells/cm^2^. The population doubling increase was calculated as explained (Cristofalo et al. 2000), using the formula *N*_H_/*N*_I_ = 2^*X*^, where *X* is the CPD (calculated as *X* = {log_10_[*N*_H_] − log_10_[*N*_I_]}/log_10_[2]), *N*_I_ is the cell inoculum number, and *N*_H_ is the VSMC harvest number.

### Gene expression analysis

Gene-specific RNA abundance was analyzed by real-time PCR in a LightCycler 1.5 using a LightCycler FastStart Master SYBR Green I kit (all from Roche, Mannheim, Germany). We used the ΔΔ*C*_T_ method of relative quantification included in the Lightcycle software, with a calibrator cDNA retrotranscribed from a combination of RNAs from aortic smooth muscle cells of 2-, 12-, and 24-month-old rats. Two endogenous reference genes were used: glyceraldehyde 3-phosphate dehydrogenase (GAPDH) for highly expressed genes such as smooth muscle markers, and acidic ribosomal protein (ARP) for moderately expressed genes, such as newly expressed bone-related RNAs. The primers used, sense (S) and antisense (AS), were the following:

Runx2: S, CTGCCGAGCTACGAAATGCC; AS, GGCCACTTGGGGAGGATTTG.
Msx2: S, ACCGAAGGGCTAAGGCAAAA; AS, CGCTGTATATGGATGCCGCT.
BMP2: S, GTTCTGTCCCTACTGATGAG; AS, ATTCGGTGCTGGAAACTAC.
MGP: S, AACACCTTTATATCCCCTCAGC; AS, GCGTTGTACCCGTAGATCAG.
Sm22*α*: S, CAGACTGTTGACCTCTTTGAAG; AS, TCTTATGCTCCTGGGCTTTC.
ARP: S, CACCTTCCCACTGGCTGAA; AS, TCCTCCGACTCTTCCTTTGC.
GAPDH: S, TCCAGTATGACTCTACCCACG; AS, CACGACATACTCAGCACCAG.

To determine the changes in protein abundance, immunoblots were performed as described in the same references (Martín-Pardillos et al. 2013, 2014) using a Trans-Blot Turbo Transfer System, and detection was performed using a VersaDoc MP 4000 System (both from Bio-Rad, Hercules, CA), after lysing the VSMC in RIPA buffer (50 mmol/L Tris pH 7.5, 150 mmol/L NaCl, 0.1% SDS, 0.5% deoxycholate, 1% Triton X-100, and a protease inhibitor cocktail, Sigma-Aldrich, St Louis, MO). Protein was quantified using a BCA kit (Pierce BCA Protein Assay; Thermo Scientific, Rockford, IL). In some cases, information was confirmed by immunofluorescence microscopy, using an Axiovert 200M fluorescent microscope equipped with Apotome (Carl Zeiss, Jena, Germany), also as reported (Martín-Pardillos et al. 2013). In this case, the exposure time was automatically adjusted to the maximal expression sample, and this time was fixed for the remaining exposures of the same antibody. The commercial antibodies used were the following: a mouse monoclonal antibody for *α*-actin (cat. A5228 from Sigma-Aldrich); mouse monoclonal antibodies anti-p21 for immunoblot (cat. sc-6246 from Santacruz, Heidelberg, Germany) and for immunofluorescence (cat. P1484, from Sigma, St. Louis, MO); a goat polyclonal anti-SM22*α* (cat. sc-18513; Santacruz); a mouse monoclonal anti-*β*-actin antibody (cat. A1978; Sigma); and a rabbit anti-phospho-p38 (cat. P1491; Sigma).

### Senescence characterization

Senescence was quantified by the activity of senescence-associated *β*-galactosidase (SA *β*-gal) at pH 6, as described (Itahana et al. 2013; Martín-Pardillos et al. 2013), using 5-bromo-4-chloro-3-indolyl-*β*-d-galactopyranoside (Sigma), after fixing the cells with paraformaldehyde.

Telomerase activity was determined by a fluorescence-based telomeric repeat amplification protocol (TRAP) using a TRAPEZE XL Telomerase Detection Kit (Millipore International, Inc., Darmstadt, Germany). Fluorescence was quantified using a DTX 880 Multimode plate reader (Beckman Coulter, Fullerton, CA).

### Calcification experiments

For calcification in vitro, quiescent VSMC in MEM were incubated with 2 mmol/L Pi for 10 days. Pi was diluted from a stock of 100 mmol/L KH_2_PO_4_/K_2_HPO4, pH 7.4. To visualize calcium deposits, cultures were stained with 0.5% alizarin red (pH 4.1–4.3) for 15 min. Calcium was quantified colorimetrically using a Calcium Assay kit (Abnoba, Taipei City, Taiwan), after overnight solubilization of calcium with 0.6 N HCl at 4°C.

### Apoptosis

Apoptosis was determined by terminal deoxynucleotidyl transferase-mediated dUTP Nick End Labeling assay, using a CLICK-IT TUNEL Alexa Fluor Imaging Assay (Molecular Probes, Eugene, OR), and visualized in an Axiovert 200M fluorescent microscope.

### Statistics

Prism 5.0 software (GraphPad Software Inc., La Jolla, CA) was used for statistics, data representation, and linear regressions. Significant differences were determined using a one-way analysis of variance and Tukey’s multiple comparison post test. Asterisks indicate significant differences when ANOVA *P*-values are <0.05 and the Tukey’s test indicates significant differences in means.

## Results

### Proliferation characteristics

**Figure 1:**
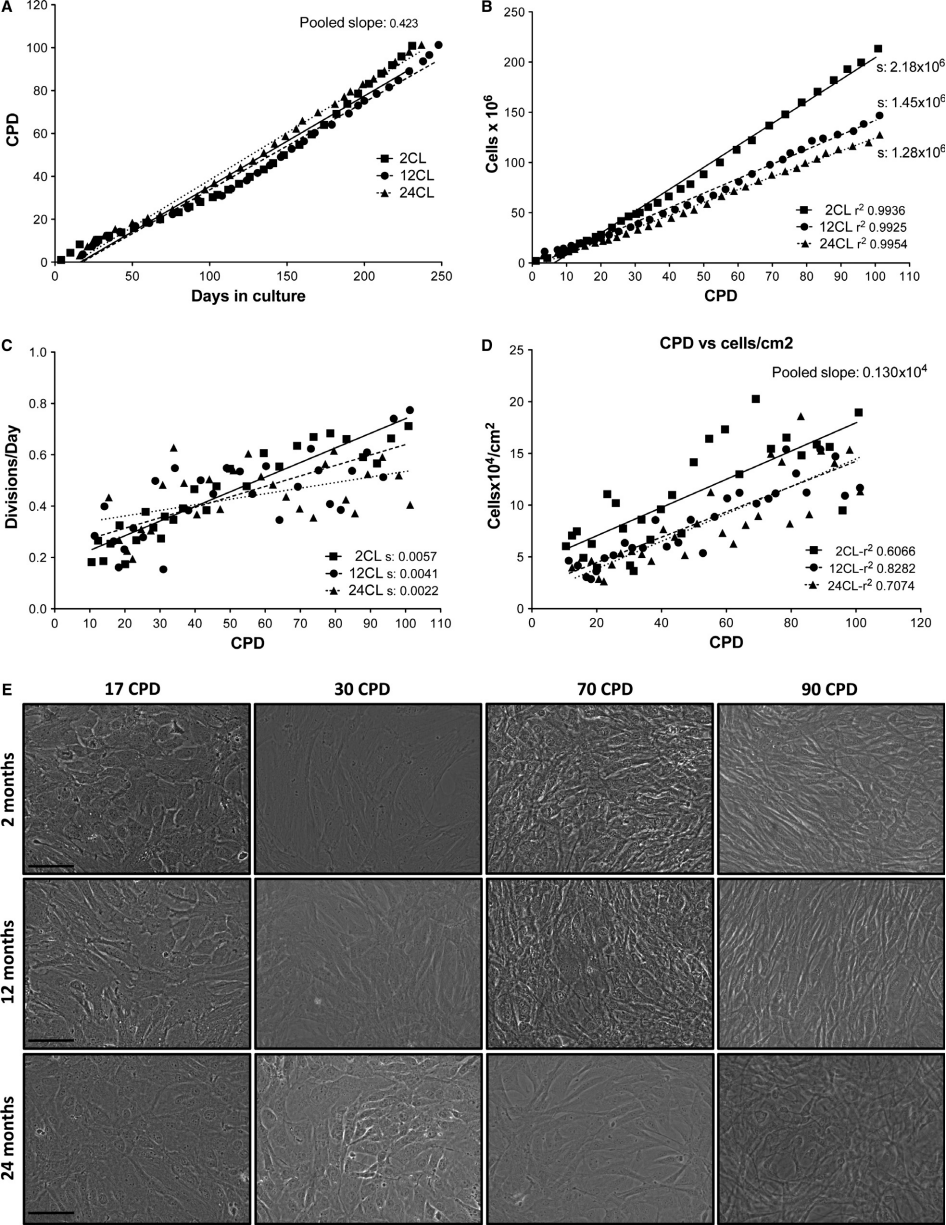
Proliferation of rat aortic smooth muscle cells from rats of 2-, 12-, and 24-month-old. (A) Cumulative population doublings (CPD) as a function of days in culture, showing no differences between the three cell lines. (B) Total number of cells grown as a function of CPD. (C) Relationships between the number of divisions per day and CPD, showing the corresponding regression lines. (D) Cell density as a function of CPD. (E) Representative microphotographs of vascular smooth muscle cells from donor rats of 2-, 12- and 24-month-old at the indicated CPD. Bar, 50 *μ*m.

Vascular smooth muscle cells were obtained from the aortas of 2-, 12-, and 24-month-old rats, which originated from the three CL of the study, namely 2CL, 12CL, and 24CL. After 17 CPD of culture stabilization, VSMC growth was characterized, with analyses performed at 17, 40, 70, and 90 CPD for all three cultures. The growth characteristics are summarized in Figure1, which shows that after 100 CPD and over 230 days in culture, the cells kept dividing, with no signs of division arrest for any of the three cultures. The relationship between CPD and days of culture were similar in all three cultures, with a pooled slope of 0.42 CPD per day (Fig.1A). Nevertheless, 2CL accumulated more cells than the 12CL and 24CL cultures, with a slope of 2.18 × 10^6^ cells per CPD, compared to 1.45 × 10^6^ and 1.28 × 10^6^ cells/CPD for 12CL and 24CL, respectively (Fig.1B). The differences between these slopes were extremely significant (*F* = 368.13, *P* < 0.0001). When we compared the divisions per day to the CPD, the differences in the slopes were very significant and followed the same trend 2CL > 12CL > 24CL (*P* = 0.00126; Fig.1C), meaning a progressive slowing down of cell division according to the donor age. Finally and surprisingly, when we compared cell density (cells per cm^2^) to the CPD, in all three CL, the density increased with the CPD at a rate of 1300 cells per CPD, given that the slopes were statistically similar (Fig.1D). The 2CL slope was parallel, but it started at a higher density.

Phase contrast microscopy (Fig.1E) revealed that the morphology of the cells changed to a longer, fibroblast-like morphology with the proliferative age (from 17 to 90 CPD), but it was apparently similar in all three donor ages. The cell number increased with the CPD, as shown in Figure1D, and the cells progressively acquired a fibroblast-like morphology, especially after 70 CPD.

### Expression of VSMC markers

**Figure 2:**
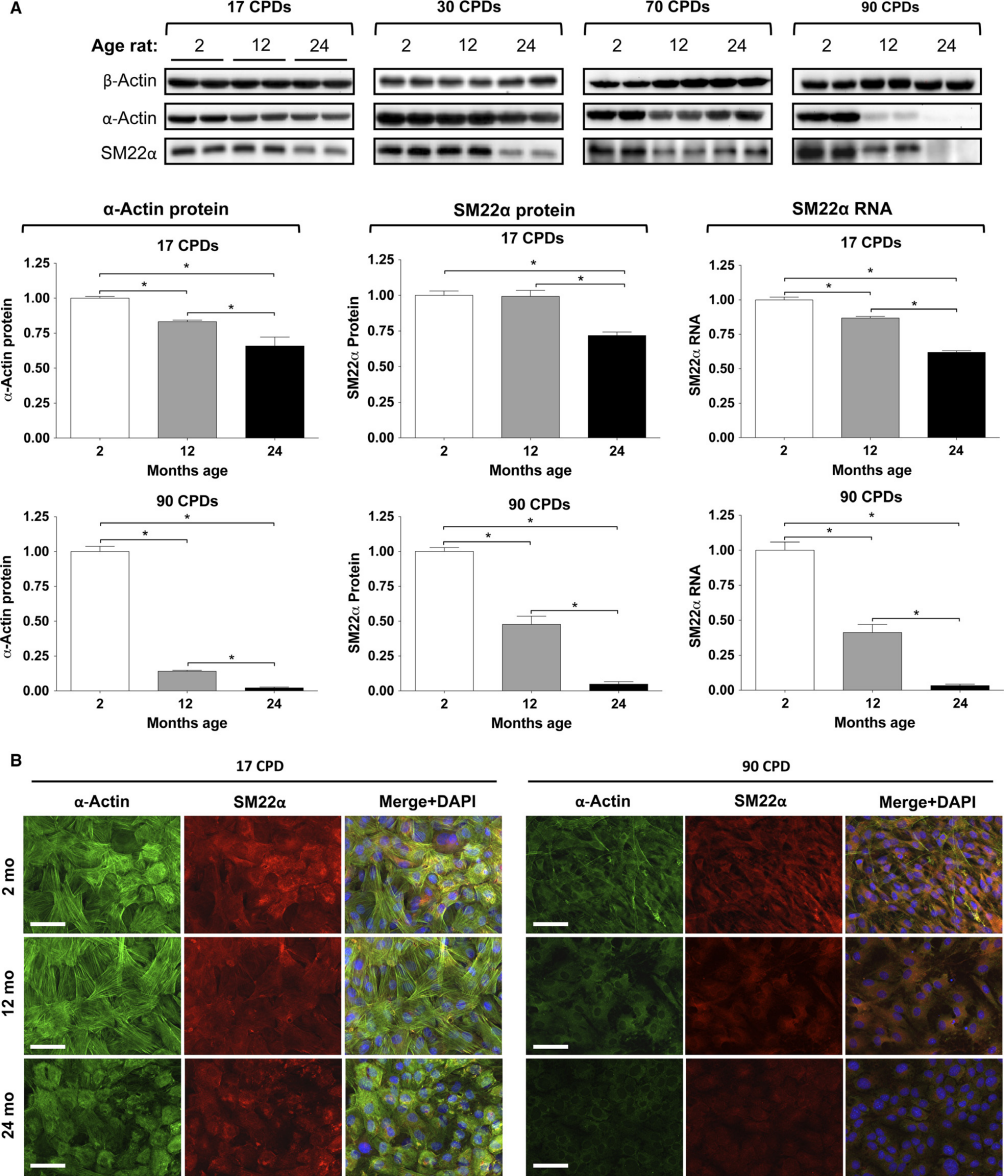
Expression of smooth muscle markers *α*-actin and SM22*α* as a function of donor age within each cumulative population doublings (CPD). (A) Immunoblot of both markers and quantitation with respect to *β*-actin expression. Only the expression quantifications at the 17th and 90th CPDs are shown. Right panels, SM22*α* RNA expression analyses by real-time PCR. (B) Immunofluorescence microphotographs of the two markers. Bar, 50 *μ*m.

We also wanted to know if the smooth muscle cell phenotype was maintained during the cultures. The contractile phenotype of VSMC was determined by analyzing the expression of the differentiation markers *α*-actin and SM22*α* in the three CL, as a function of both the proliferative and the chronological ages. Figure2 shows the effect of donor (chronological) age by comparing the expression of both VSMC markers (plus *β*-actin as a control) in 2CL, 12CL, and 24CL within each CPD group, and it shows the quantitative densitometry of the bands for 17 and 90 CPD. The abundance of both markers was slightly, but significantly lower according to the donor age in all three CL. The expression of *α*-actin in 12CL and 24CL was 0.8 and 0.7 times less, respectively, when compared to 2CL at 17 CPD. SM22*α*, however, was only lower in 24CL (0.7 times less compared to 2CL). The difference of the expression in 12CL and 24CL with respect to 2CL increased dramatically with the CPD, such that the expression of *α*-actin in 12CL at 90 CPD was 0.2 times less that of 2CL, and it was hardly visible in 24CL. A similar pattern was observed for the abundance of SM22*α* protein and RNA. These changes in protein expression were confirmed by immunofluorescence microscopy (Fig.2B).

**Figure 3:**
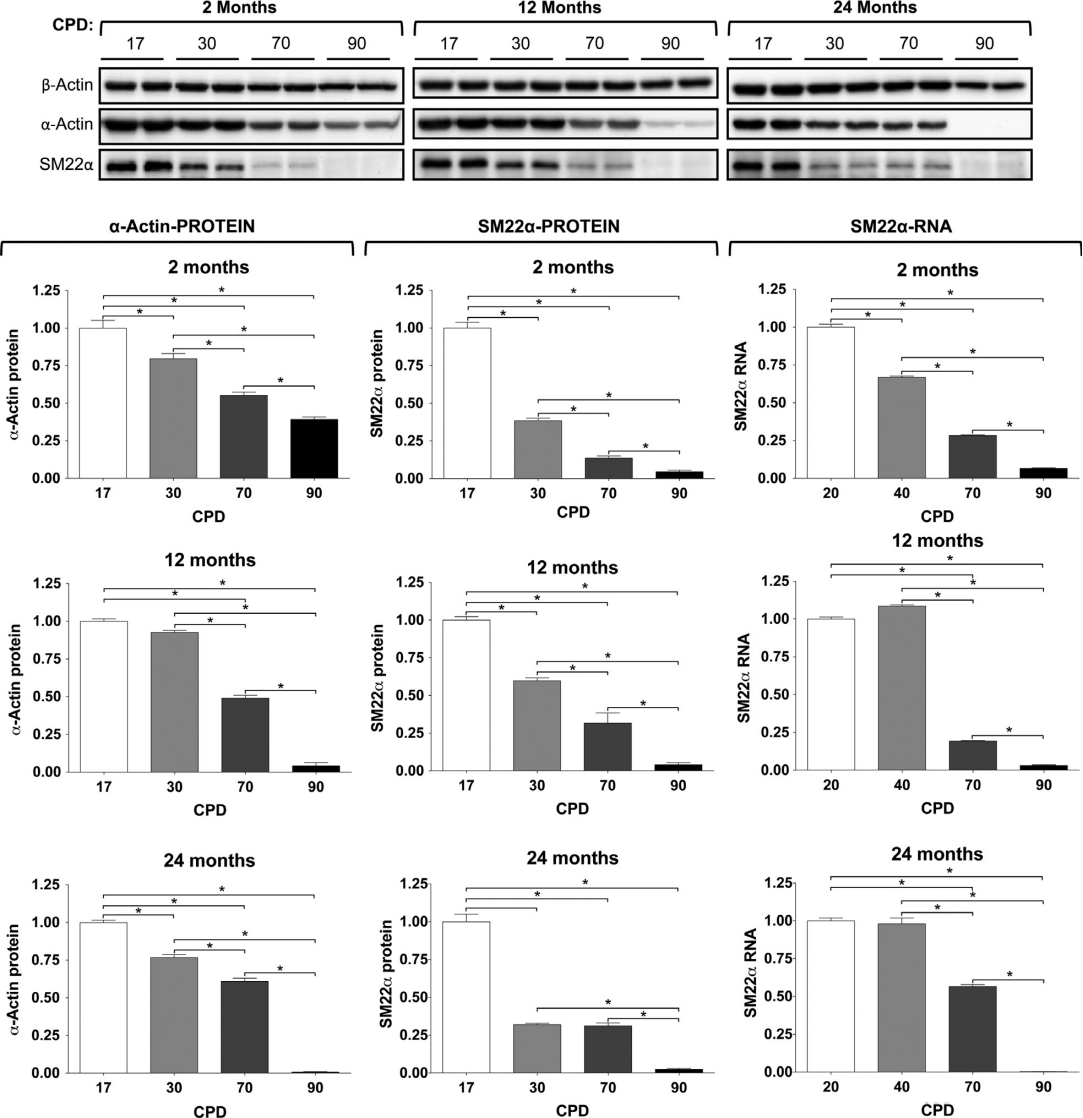
Expression of smooth muscle markers *α*-actin and SM22*α* as a function cumulative population doublings (CPD) within each donor age. Top, immunoblot of both markers plus *β*-actin. Bottom, densitometric quantitation of the signals in immunoblots and SM22*α* RNA quantitation.

Changes in *α*-actin and SM22*α* expressions as a function of the proliferative age of the three CL are compiled in Figure3. The results confirm that the expression of both VSMC markers diminished considerably as the CPD increased, and it even disappeared at 90 CPD of 12CL and 24CL. These findings were also confirmed by microscopy (data not shown).

### Senescence of VSMC

**Figure 4:**
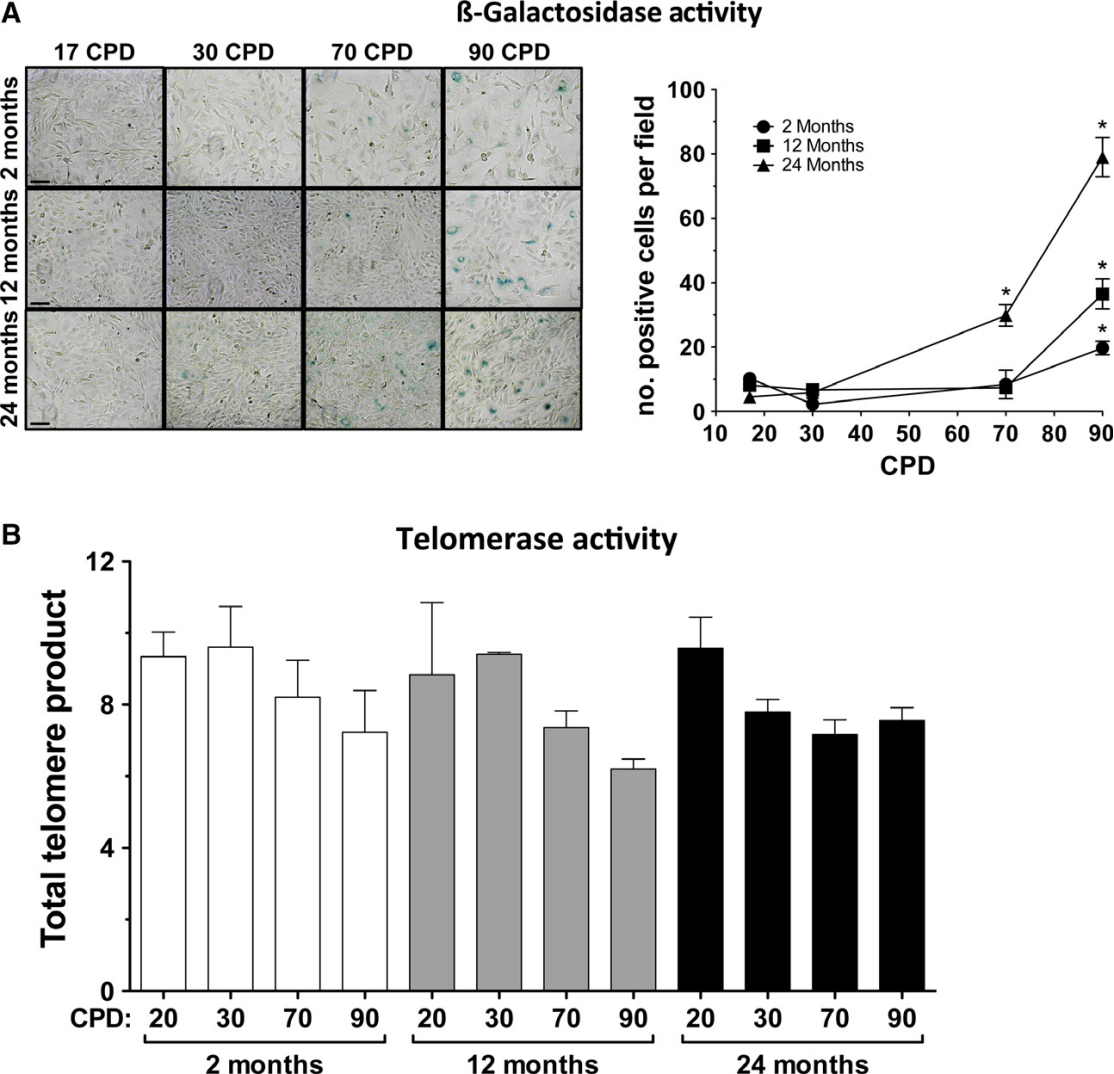
Characterization of cell senescence in vascular smooth muscle cells (VSMC). (A) Microphotographs of VSMC stained for *β*-galactosidase activity. Bar, 100 *μ*m. To the right of the pictures, chart of the means of *β*-galactosidase-positive cells per microscopy field at 100 times magnification. (B) Telomerase activity as a function of donor age and cumulative population doublings (CPD).

Although the cells did not arrest due to proliferation even after 100 CPD, we checked for the presence of senescence markers, such as *β*-galactosidase and telomerase activity. For senescence-associated *β*-galactosidase activity, the corresponding staining was done using VSMC from 2CL, 12CL, and 24CL, measured at 17, 30, 70, and 90 CPD. The representative images are shown in Figure4A, thus showing the mean number of cells per microscopy field at 100×. The cells from 2- and 12-month-old rats only showed a clear increase in staining at 90 CPD. The cells from 24-month-old rats, however, also showed a minimal, but evident increase at 70 CPD.

The effects of donor age and proliferation did not have significant effects on telomerase activity. These results are summarized in Figure4B, which shows that in all three CL the total telomere product was similar, with a trend of decreasing activity as the CPD increased, but the changes were not statistically different.

### Apoptosis

**Figure 5:**
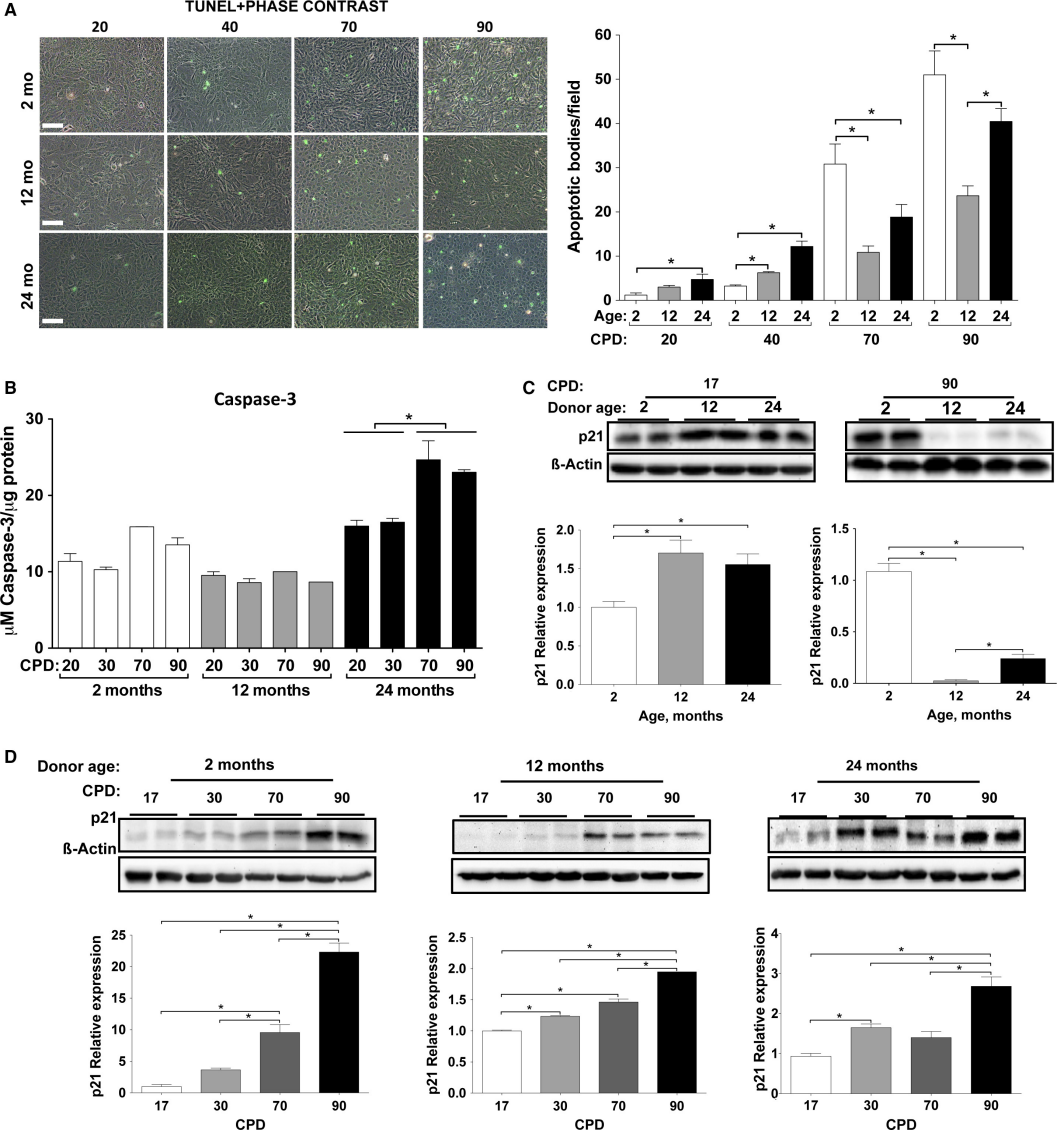
Analysis of apoptosis in cultured vascular smooth muscle cells (VSMC). (A) Microphotographs of VSMC stained with fluorescent TUNEL as a function cumulative population doublings (CPD) from 2-, 12-, and 24-month-old rats and merged with phase contrast to show all present cells. Bar, 100 *μ*m. The frequency of TUNEL-positive nuclei after counting in microscope fields is shown to the right. (B) Caspase-3 activity as a function of donor age and CPD. (C) Expression of p21 as a function of donor age at 17 and 90 CPD. (D) Expression of p21 as a function of donor CPD in 2-, 12-, and 24-month-old rats. Histograms, quantification of western blot signal densities with respect to *β*-actin.

The effect of CPD and donor age on the apoptosis rate of VSMC was studied by conducting a TUNEL assay and an analysis of caspase-3 activity (Fig.5). Fluorescent TUNEL revealed that apoptotic cells increased with both CPD and rat age within each CPD, which was confirmed by counting the apoptotic cells in four different centered fields of each experimental condition (donor age and CPD). The apoptosis increase was biochemically confirmed by measuring caspase-3 activity (Fig.5B), but in this case, the increase was only observed in VSMC from 24-month-old rats, compared to the VSMC from 2-month-old rats. The increase in caspase-3 activity was also observed with respect to CPD, especially at 70 and 90 CPD in 2CL and 24CL.

p21^Cip1^ (cyclin-dependent kinase inhibitor 1) expression and its accumulation with CPD were analyzed by western blot because this expression is related to apoptosis and senescence (cell cycle arrest). p21 expression at early CPD (17 and 30 CPD) increased with the age of the animal, while at 70 and 90 CPD, p21 expression nearly disappeared in the VSMC from 12- and 24-month-old animals (Fig.5C). When the expression of p21 was correlated with CPD, p21 expression increased with CPD in 12CL and 24CL, even if the cells did not stop dividing (Fig.5D).

### Oxidative stress

**Figure 6:**
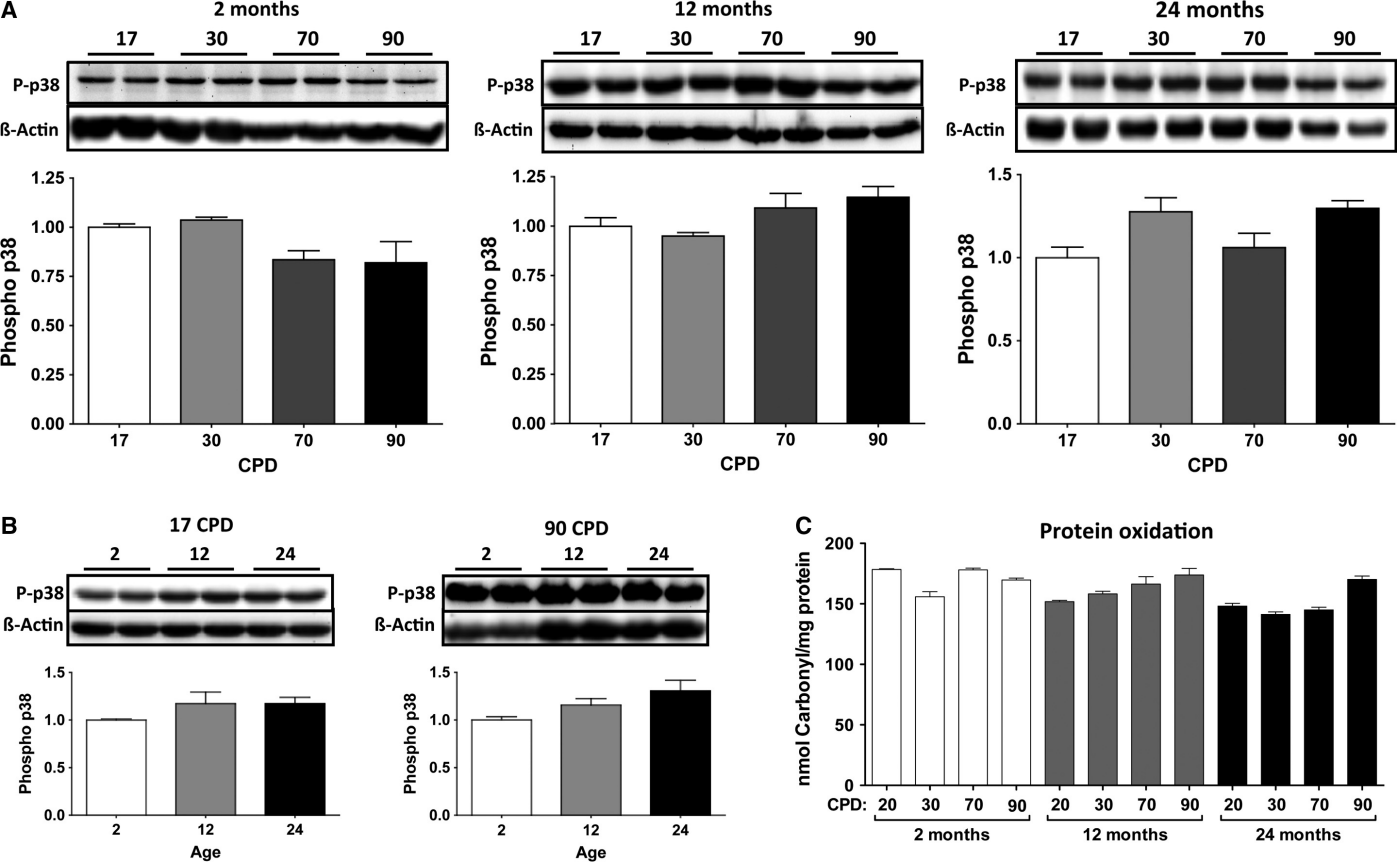
Analysis of oxidative stress as a function of donor age and cumulative population doublings (CPD). (A) Expression of phosphorylated p38 as a function of CPD in the three donor ages. (B) Expression of phospho-p38 as a function of donor age at 17 and 90 CPD. 30 and 70 CPD also did not show effects on p38 expression. (C) Analysis of protein oxidation as molar carbonyl content, as a function of donor age and CPD.

Aging-related oxidative stress was analyzed in the three CL as a function of donor age and proliferative lifespan. First, we analyzed the expression of phospho-p38, a MAP kinase activated by several cell stresses, including the oxidation increase that occurs in aging (Ragnauth et al. 2010; Gutiérrez-Uzquiza et al. 2012). Figure6 shows that the expression of stress-response protein phospho-p38 did not significantly change with the CPD in any of the three CL (A) or as a function of donor age (B). To confirm that the absence of cell stress evidence also meant the absence of oxidative damage accumulation, the protein carbonyl content was also analyzed, thereby revealing no changes in the oxidative status of the cells, either as a function of donor age or proliferative age (Fig.6C).

### Osteogene expression

**Figure 7:**
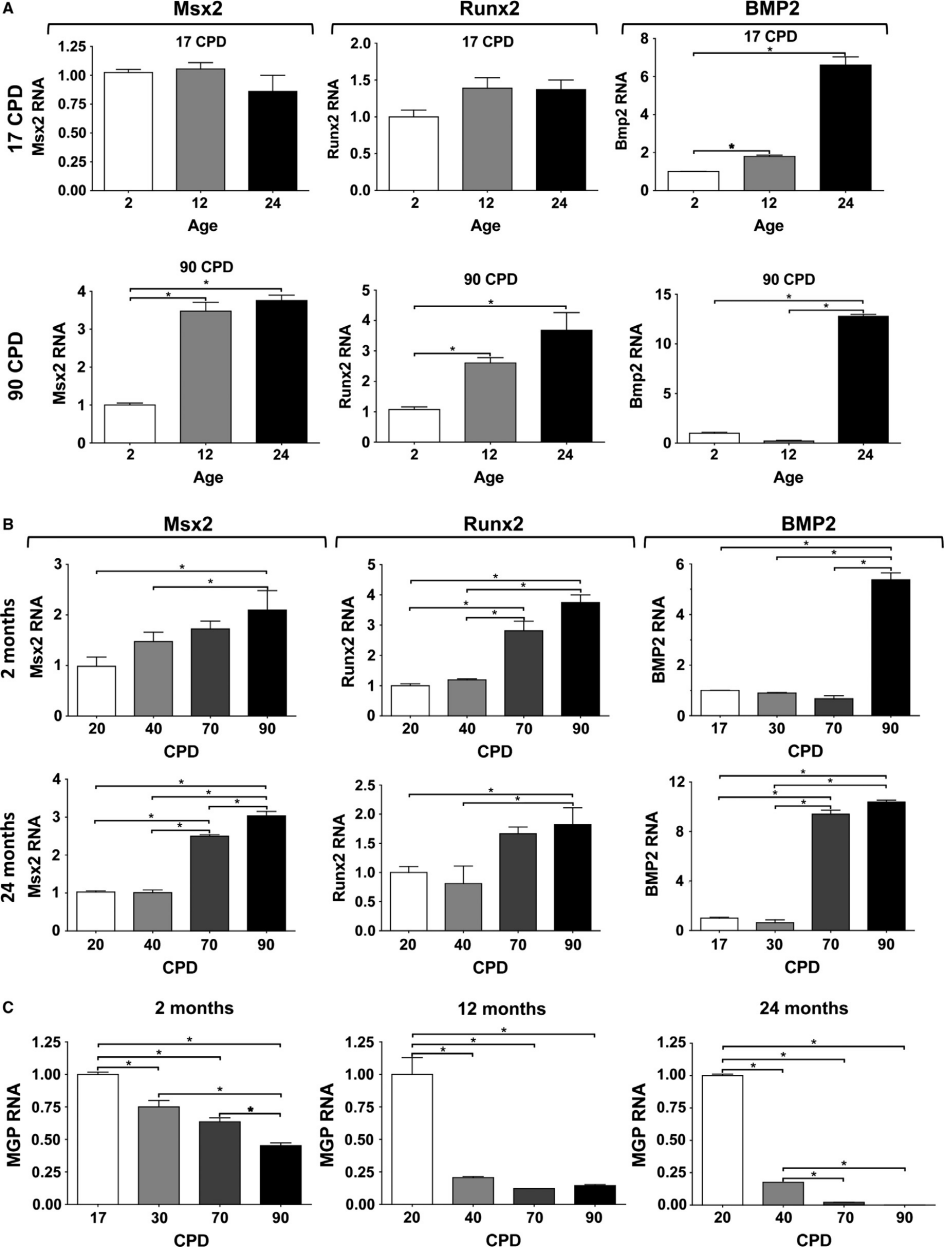
Effect of age and cumulative population doublings (CPD) on osteogene expression and *trans*differentiation. (A) Expression of Msx2, Runx2, and Bmp2 as a function of donor age at 17 and 90 CPD. (B) Expression as a function of CPD and different donor ages. (C) Expression of MGP as a function of CPD at the three donor ages.

RNA was obtained from the cells of 2CL, 12CL, and 24CL maintained in regular MEM and at the same CPD as above. The basal expressions of the Msx2, Runx2, and Bmp2 osteogene RNAs were analyzed by real-time PCR. The results are summarized in Figure7. The effect of donor age on osteogene expression is shown for 17 and 90 CPD. It was found that the Msx2 and Runx2 expressions were similar in the three CL at 17 CPD, but these expressions considerably increased with age at 90 CPD. Bmp2 abundance, however, increased considerably in 24CL at all CPDs.

An analysis of relative expression as a function of CPD revealed that all Msx2 and Runx2 expressions increased with the CPD, while Bmp2 showed a dramatic increase at an advanced CPD (Fig.7B). The increase was similar in all three CL.

Finally, the RNA expression of a major anticalcifying protein, MGP, was also analyzed in the same samples, revealing a decrease in expression as donor age and proliferative age increased in all three CL. Figure7C shows the expression of MGP as a function of CPD for the three donor ages.

### Response to 2 mmol/L Pi treatment

**Figure 8:**
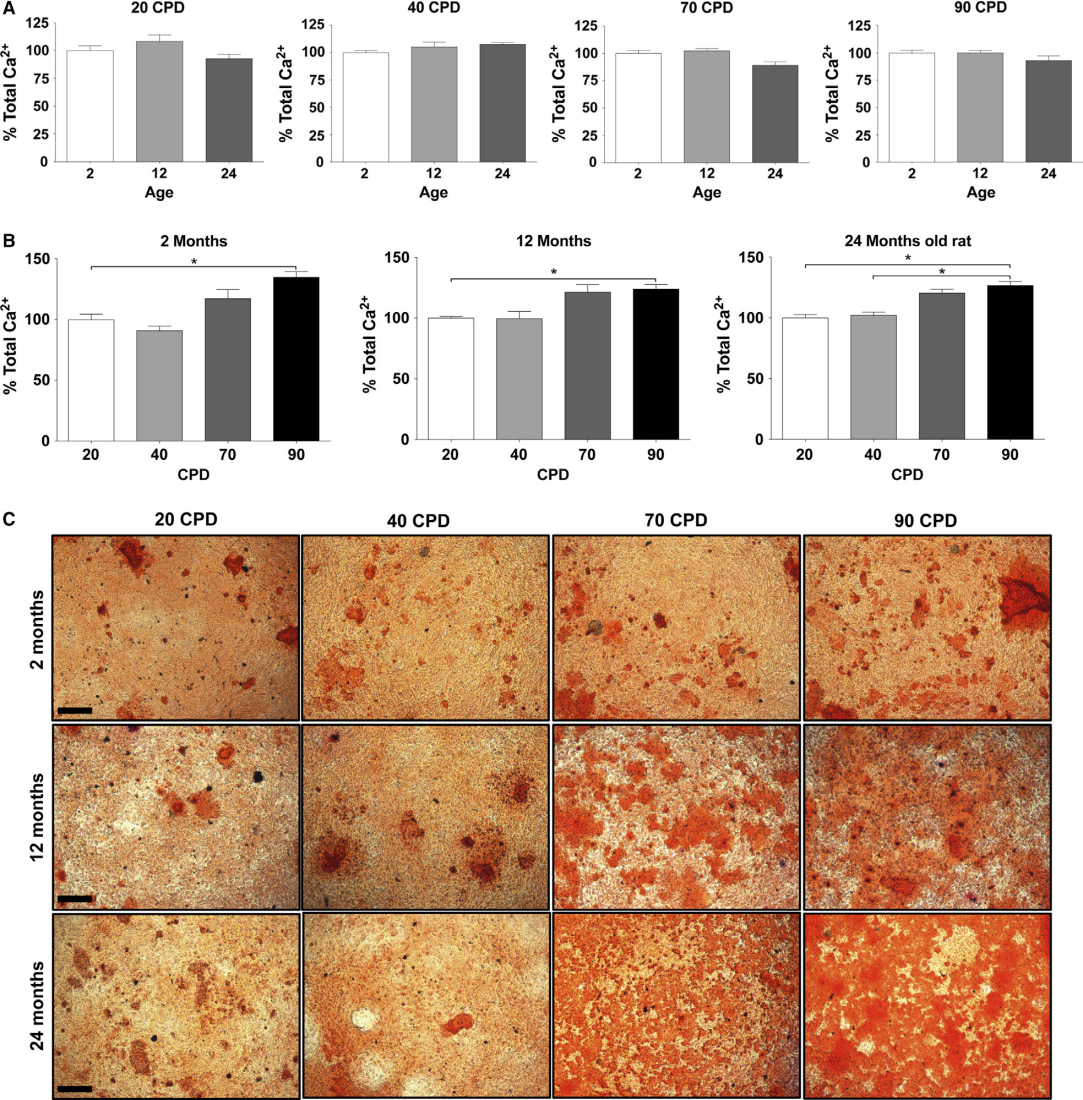
Effect of age and proliferation on the calcium deposition caused by 2 mmo/L Pi in minimum essential medium. (A) Effect of donor age at all four cumulative population doublings (CPDs). (B) Effect of CPD at all three donor ages. (C) Alizarin staining of calcium deposits of the indicated experimental conditions. Bar, 200 *μ*m.

Vascular smooth muscle cells from the three CL and four CPDs were incubated in MEM with 2 mmol/L Pi for 10 days to study the response of the cells to a classical calcification medium. Staining with alizarin red revealed that calcium deposition increased with the CPD, but the donor age variable had no effect on the deposited calcium at any CPD (Fig.8A). These data were confirmed with alizarin red staining (Fig.8B).

## Discussion

Medial VC is extremely common in the elderly (Blumenthal et al. 1944). However, while the hemodynamic complications are similar to those that affect renal patients, the outcomes are usually less severe (Roosens et al. 2012), as evidenced by the fact that MVC is usually accidentally described in the elderly population when diagnosing other disorders. During the last decade, many studies have addressed several aspects of arterial smooth muscle cell aging, and they show a correlation between VSMC senescence and age-related arterial dysfunctions. This correlation has been made based on the assumption that proliferative aging (in vitro) is similar to cell aging in vivo (Hayflick 1965; Campisi 2013). Studies have revealed, for example, that human VSMC aged in vitro showed an increase in the expression of senescence markers such as p16 and p21 and an increase in senescence-associated *β*-galactosidase activity, as well as an increase in the abundance of several bone-related genes such as alkaline phosphatase, Runx2, or collagen I, in addition to a reduction in MGP expression (Nakano-Kurimoto et al. 2009). Bmp2 and Msx2 expressions were not modified by replicative senescence, and osterix was somewhat less. A concurrent study, which also analyzed the human senescent VSMC gene expression profile using the microarray approach, expanded the analysis to inflammation and tissue remodeling genes (Burton et al. 2010). The study found overexpression of Bmp2 in senescent cells and the inhibition of MGP and osteoprotegerin, among other genes. Nevertheless, gene expression changes do not occur exclusively with senescence, and VSMC cultures dramatically change the expressed genome according to the proliferative activity and confluence (Absher et al. 1989; Shanahan et al. 1993), thereby making it more difficult to compare in vivo and in vitro VSMC phenotypes.

In this work, we have used primary cultures of rat VSMC, because laboratory rats, like humans, show increasing medial VC with age (Roosens et al. 2012). We have studied the age-related changes of VSMC regarding the two age concepts: proliferative aging (in vitro) and chronological aging (in vivo). To do so, we established three CL of VSMC obtained from groups of rats that were 2-, 12-, and 24-months-old, which led to longitudinal and transversal studies. First, we summarized the culture growth characteristics of the three CL (Fig.1), with the remarkable finding that no signs of proliferative aging or senescence were observed, even after 100 CPD. Only a reduction in the total number of cells was observed in 12CL and 24CL (Fig.1B), as well as a progressive reduction in the cell divisions per day according to the CPD (Fig.1C), compared to 2CL. The limited effects on rat VSMC proliferation are not common to all species, for example, human VSMC show a reduced cell division rate as donor age increases (Bierman 1978; Ruiz-Torres et al. 1999). The absence of cell senescence was corroborated by staining for *β*-galactosidase activity (with the exception of a slight increase at 90 CPD in VSMC from 24-month-old rats) and a constant telomerase rate, which agrees with previous findings that report telomerase induction during rat VSMC proliferation (Fig.4) (Minamino and Kourembanas 2001). In addition, the absence of apparent oxidative stress (Fig.6) also agrees with the absence of senescence because oxidative stress is a known cause of the senescence-associated secretory phenotype, DNA damage and growth arrest, and telomere shortening (Campisi 2013). We used p38 activation as a marker of several stresses, even though this MAK kinase has been traditionally used as evidence of oxidative damage (Ragnauth et al. 2010; Gutiérrez-Uzquiza et al. 2012). The absence of phospho-p38 overexpression, therefore suggests that not only was oxidative stress not present in the cells, but also no other mechanisms of stress and stimuli that could activate this MAP kinase (osmostress, ultraviolet light, heat shock, cytokines, etc.) were significantly affecting the cultures (de Nadal et al. 2011). Rather than senescence, we observed a clearly increasing rate of VSMC apoptosis as the CPD increased (Fig.5). A TUNEL assay revealed that, in addition to the effect of CPD in vitro, apoptosis also increased with donor age. However, this effect is only clearly observed at early CPD because at 70 and 90 CPD, the VSMC from 2CL showed the highest apoptosis rate. While the findings were less evident when caspase-3 activity was determined, this assay corroborated the trend of an increasing apoptotic rate with the increase in both proliferative and donor aging.

Given that the VSMC phenotype changes easily from the synthetic (proliferating) to the contractile states (Absher et al. 1989; Shanahan et al. 1993) in vitro, which also seems to occur in vivo under certain circumstances, we studied the expression of the VSMC phenotype during the proliferative life in the three CL. The analyses of the expression of aortic smooth muscle markers, such as *α*-actin and SM22*α*, revealed a significant effect by donor age, which was observed as early as 17 CPD and was maximal at 90 CPD (Fig.2). The effect of proliferative aging was similar, but stronger than the effect of chronological aging (Fig.3), and the effect increased as donor age increased. Consequently, at the same time as this apparent dedifferentiation, we also studied the conversion of VSMC into an osteoblastic phenotype by measuring the RNA expression of the Msx2, Runx2, and Bmp2 osteogenes (Fig.7). Our results agreed with previous reports (Nakano-Kurimoto et al. 2009; Burton et al. 2010) and showed evidence of osteoblastic transdifferentiation with the progression of the proliferative life in all three CL. The effect of donor age was, however, much more limited. At early CPD, only Bmp2 clearly increased in 24CL, but at late CPD, all three osteogenes increased strongly in 12CL and 24CL, compared to 2CL. Conversely, the expression of the anticalcifying protein MGP decreased with both donor age and proliferative age.

Finally, even if the incubation of VSMC at a high Pi concentration is a very limited model of calcification in vitro, with biological and chemical artifacts (Hortells, Sosa, Millan, and Sorribas, manuscript under review), the use of this protocol with the three CL and four CPDs has provided findings that are consistent with the gene expression results: calcium deposition is not affected by donor age, but CPD has a moderate effect when VSMC are incubated with 2 mmol/L Pi in MEM for 10 days (Fig.8).

We must stress that this study was limited to a comparison of the effects of donor age with the effects of proliferation age, wherefore all the experiments were limited to in vitro conditions. If a comparison is made directly between the phenotypic changes observed in vitro and the chronological aging of arteries in vivo, the magnitude of the observed differences would be greater. The reason is because the existence of arteries without contractile VSMC (i.e., cells without smooth muscle markers such as *α*-actin or Sm22*α*, as observed at 90 CPD, Fig.3) has not been observed in the elderly, and in fact, this *trans*differentiation would not be compatible with life. Based on these relevant differences, the critical steps and conditions of the method of VSMC aging in vitro should be analyzed in detail. During a study of VSMC aging in vitro, there are two critical steps that affect the VSMC phenotype and survival selection: (1) the isolation of cells from the artery, followed by the establishment of a culture, and (2) the culture conditions and techniques during proliferative aging. First, the isolation of VSMC by the collagenase digestion of arteries and seeding is a very aggressive method of cell selection. This selection occurs mostly according to the state of health of the cells and also partly according to the cell type. Moreover, the fact that fibroblasts could express *α*-actin upon isolation and culture is an indication of possible cell contamination (e.g., Masur et al. 1996). During proliferation, either after the first seeding or after every trypsinization, the VSMC phenotype changes dramatically from the contractile to the proliferating phenotype (Shanahan et al. 1993; Shanahan and Weissberg 1998; Liu et al. 2015). After several passages and population doublings, this plasticity is reduced in favor of cells with a fibroblast-like morphology, which can be observed in Figure1E and which is corroborated by the dedifferentiation shown in Figures2 and 3 (Absher et al. 1989). The apparent changes in gene expression during a long-term culture can be caused by clonal selections during trypsinizations and forced proliferation if the clones exhibit different growth rates, but they can also be caused by the composition of the culture media, the plastic support, the CO_2_ atmosphere, culture manipulations, pH fluctuations, etc. All of these stressing factors that do not occur in vivo can be affecting the type of cells that survive in a culture during passages and divisions, and therefore the relationship with the aging process in vivo is questionable.

In conclusion, our findings reveal that, in vitro*,* rat VSMC do not exhibit classical cell division arrest or senescence (most likely because telomerase is induced) or an increasingly oxidative steady state, which is a hallmark of aging. We have also observed the previously reported partial osteoblastic transition of VSMC that occurs during the proliferative lifespan. However, given that senescence in vitro does not take place and that no other characteristics of aging were observed in the CL, special caution should be observed when using the proliferative lifespan of VSMC as a model of aging in vivo, as explained above. For example, despite osteoblastic transition in vitro, this transition has not been observed, or only minimally, as a function of donor age. The same can be said about maintenance of the contractile phenotype, for which CPD has a stronger effect than donor age.

We do not know the point up to which the proliferative lifespan of VSMC represents aging in vivo, but at a minimum, the effects on osteoblastic transdifferentiation are exaggerated in comparison to aging in vivo, and some hallmarks of aging, such as oxidation product accumulation, are not observed, despite the continuous elimination of defective cells during proliferation. Therefore, any conclusion achieved using this in vitro model of aging should be corroborated using arteries of old animals.

## Conflict of Interest

None declared.
